# Overview of the RGD-Based PET Agents Use in Patients With Cardiovascular Diseases: A Systematic Review

**DOI:** 10.3389/fmed.2022.887508

**Published:** 2022-05-06

**Authors:** Matthieu Dietz, Christel H. Kamani, Vincent Dunet, Stephane Fournier, Vladimir Rubimbura, Nathalie Testart Dardel, Ana Schaefer, Mario Jreige, Sarah Boughdad, Marie Nicod Lalonde, Niklaus Schaefer, Nathan Mewton, John O. Prior, Giorgio Treglia

**Affiliations:** ^1^Nuclear Medicine and Molecular Imaging Department, Lausanne University Hospital, Lausanne, Switzerland; ^2^INSERM U1060, CarMeN Laboratory, University of Lyon, Lyon, France; ^3^Cardiology Department, Lausanne University Hospital, Lausanne, Switzerland; ^4^Department of Diagnostic and Interventional Radiology, Lausanne University Hospital, Lausanne, Switzerland; ^5^University of Lausanne, Lausanne, Switzerland; ^6^Cardiovascular Hospital Louis Pradel, Department of Heart Failure, Hospices Civils de Lyon, Lyon, France; ^7^Clinical Investigation Center Inserm 1407, Lyon, France; ^8^Clinic of Nuclear Medicine, Imaging Institute of Southern Switzerland, Ente Ospedaliero Cantonale, Bellinzona, Switzerland; ^9^Università della Svizzera Italiana, Lugano, Switzerland

**Keywords:** αvβ_3_ integrin, RGD, angiogenesis, positron emission tomography, cardiovascular diseases, myocardial infarction, atherosclerosis

## Abstract

Studies using arginine–glycine–aspartate (RGD)-PET agents in cardiovascular diseases have been recently published. The aim of this systematic review was to perform an updated, evidence-based summary about the role of RGD-based PET agents in patients with cardiovascular diseases to better address future research in this setting. Original articles within the field of interest reporting the role of RGD-based PET agents in patients with cardiovascular diseases were eligible for inclusion in this systematic review. A systematic literature search of PubMed/MEDLINE and Cochrane library databases was performed until October 26, 2021. Literature shows an increasing role of RGD-based PET agents in patients with cardiovascular diseases. Overall, two main topics emerged: the infarcted myocardium and atherosclerosis. The existing studies support that α_v_β_3_ integrin expression in the infarcted myocardium is well evident in RGD PET/CT scans. RGD-based PET radiotracers accumulate at the site of infarction as early as 3 days and seem to be peaking at 1–3 weeks post myocardial infarction before decreasing, but only 1 study assessed serial changes of myocardial RGD-based PET uptake after ischemic events. RGD-based PET uptake in large vessels showed correlation with CT plaque burden, and increased signal was found in patients with prior cardiovascular events. In human atherosclerotic carotid plaques, increased PET signal was observed in stenotic compared with non-stenotic areas based on MR or CT angiography data. Histopathological analysis found a co-localization between tracer accumulation and areas of α_v_β_3_ expression. Promising applications using RGD-based PET agents are emerging, such as prediction of remodeling processes in the infarcted myocardium or detection of active atherosclerosis, with potentially significant clinical impact.

## Introduction

Ischemic heart disease is the leading cause of death worldwide (1). Understanding cellular alterations involved in atherosclerotic plaque instability, as well as reparative mechanisms following myocardial infarction (MI) is important for both preventive and therapeutic intervention.

Positron emission tomography (PET) is an *in vivo* medical imaging technique that enables quantification of radiotracer uptake in the entire body at a cellular level.

Integrin α_v_β_3_ is a transmembrane receptor mediating cell adhesion that influences cell growth, proliferation, survival, and migration. The peptide motif arginine–glycine–aspartate, abbreviated by “RGD” in the one-letter code, has been identified in 1984 as a minimal amino acid sequence that some integrins recognize in their natural ligands (2). In 1991 a group from Germany reported a strong and selective binding of cyclic pentapeptides containing the RGD sequence to α_v_β_3_ integrin (3). The past 20 years have witnessed a remarkable expansion on cyclic peptides containing the RGD sequence since it was found that α_v_β_3_ integrin plays a major role in angiogenesis, further enhanced by the first successful clinical applications of the PET radiopharmaceutical ^18^F-Galacto-RGD (4–8).

The integrin-mediated cell adhesion/migration imaging with RGD PET was found to be involved in a wide range of pathophysiological processes, not only related to angiogenesis. Increased α_v_β_3_ expression has been observed in some cancer cells as well as in cells involved in extracellular matrix remodeling such as fibroblasts and activated macrophages (8, 9). However, the clinical relevance of such biomarker is not well understood (8). Although most studies using RGD-PET agents focused predominantly on tumorigenesis, a greater focus on non-oncological applications such as cardiovascular diseases is of interest (9). In fact, in the development of atherosclerosis, expression of integrin α_v_β_3_ has been found in endothelial cells as well as in CD68-positive macrophages, which are known as key factors in plaque instability (10–12). Moreover, α_v_β_3_ integrin expression seems to appear central to the coordination of myocardial repair following MI. α_v_β_3_ integrin is indeed upregulated in states of angiogenesis within the infarcted myocardium and could be expressed by activated myofibroblasts and macrophages during margination and chemotaxis (13, 14).

Some recent studies using RGD-PET agents in cardiovascular diseases have been published. The aim of this systematic review is to perform an updated evidence-based summary about the role of RGD-based PET agents in patients with cardiovascular diseases to better address further research in this setting.

## Materials and Methods

The reporting of this systematic review conforms to the updated “Preferred Reporting Items for a Systematic Review and Meta-Analysis” (PRISMA) statement, an established guidance to identify, select, appraise, and synthesize studies in systematic reviews (15).

### Search Strategy

A comprehensive computer literature search of PubMed/MEDLINE, and Cochrane library databases was performed by two authors (MD and GT) to identify published articles that investigated the role of RGD-based PET agents in patients with cardiovascular diseases. A combination of the following terms was used for the search algorithm: [(integrin) OR (angiogenesis) OR (RGD) OR (NODAGA)] AND [(PET) OR (positron)] AND ((myocard^*^) OR (cardi^*^) OR (heart) OR (cardiovascular) OR (CAD)]. The search was carried out from inception to October 26, 2021. To expand the search, references of the retrieved articles were also screened for additional studies.

### Study Selection

Original articles within the field of interest reporting the role of RGD-based PET agents in patients with cardiovascular diseases were eligible for inclusion. The exclusion criteria were as follow: (a) original preclinical studies in the field of interest; (b) articles outside of the field of interest of this review; (c) case reports and small case series (<5 patients); (d) review articles, comments, letters, editorials, and conference proceedings. No language or date restrictions were used. The titles and abstracts of the recovered articles were reviewed independently by two researchers (MD and GT) according to the inclusion and exclusion criteria. Articles which appeared evidently ineligible according to these eligibility criteria were rejected. The full-length version of the remaining articles was independently reviewed by two researchers (MD and GT) to evaluate their eligibility for inclusion. Any disagreements over articles eligibility were resolved by consensus.

### Quality Assessment

The quality assessment was performed according to the NIH Quality assessment tool for Observational Cohort and Cross-Sectional Studies (https://www.nhlbi.nih.gov/health-topics/study-quality-assessment-tools).

## Results

### Literature Search

**Figure 1:**
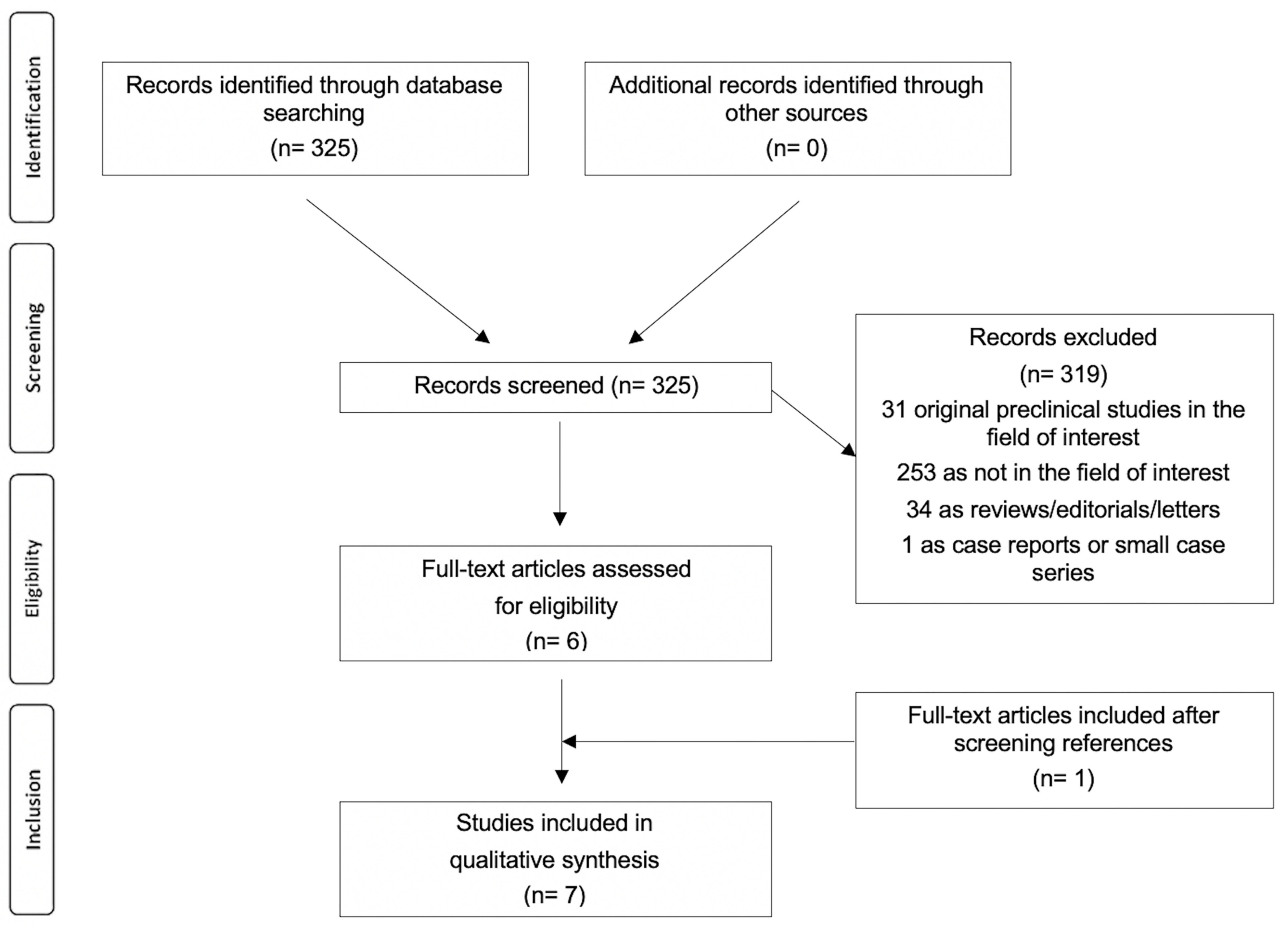
Flowchart of the literature search.

The review question was the role of RGD-based PET agents in patients with cardiovascular diseases. The literature search results using a systematic approach are reported in Figure 1. The comprehensive computer literature search from PubMed/MEDLINE and Cochrane library database revealed 325 records. Reviewing titles and abstracts, 319 records were excluded: 253 because they were not in the field of interest of this review; 34 reviews, editorials, letters, or comments; 31 preclinical studies, and 1 case report. Six articles were selected and retrieved in full-text version. One additional study was found screening the references of the selected articles. Finally, 7 articles (198 patients) including data on the role of RGD-based PET agents in patients with cardiovascular diseases were included in the systematic review (16–22). The characteristics of the studies selected for the systematic review are presented in Tables 1–5.

**Table 1:** Basic study and patient characteristics of the included studies.

**References**	**Country**	**Study design**		**Study focus**	**Number of patients**	**MI patients**	**Stroke patients or patients scheduled for carotid endarterectomy**	**Average age (range)**	**Male/** **Female**
Choi et al. (22)	Korea	Prospective	Monocentric	Stroke	10	NR	10	6.1 (2–14)*	5/5^†^
Sun et al. (16)	China	Prospective	Monocentric	MI	39	23	16	MI Patients: 61 (45–82) Stroke Patients: 56 (33–80)	30/9
Beer et al. (17)	Germany	Prospective	Monocentric	Atherosclerosis	10	NR	10	68.5 (55–79)	NR
Jenkins et al. (19)	United Kingdom	Prospective	Monocentric	MI	37	21	NR	64 ± 10 (NR)	27/10
Jenkins et al. (19)	United Kingdom	Prospective	Monocentric	Atherosclerosis	46	24	NR	66 ± 10 (NR)	34/12
Makowski et al. (20)	Germany	Prospective	Monocentric	MI	12	12	NR	53 ± 12 (NR)	12/0
Dietz et al. (21)	Switzerland	Retrospective	Monocentric	Atherosclerosis	44	5	5	60 (NR)	24/20

*MI, myocardial infarction; NR, not reported.*

^*^Age 1–14 years in the method section.

*^†^Male/Female 6/4 in the method section*.

**Table 2:** Technical aspects of RGD-based PET/CT in the included studies.

**References**	**PET/CT scanner**	**Tracers used**	**Mean injected activity (range)**	**Mean time interval between radiotracer injection and image acquisition**	**PET/CT image analysis**	
					**Semi-quantative**	**Reference**
Choi et al. (22)	NR	^68^Ga-NOTA-RGD	111	20 min	Lesion to control ratios	Normal brain
Sun et al. (16)	Siemens Biograph 64 TruepointTrueV	^68^Ga-NOTA-PRGD2	1.85 MBq/kg	30 min	SUV_peak_; SUV ratios	Normal myocardium; normal brain
Beer et al. (17)	Siemens Biograph Sensation 16	^18^F-Galacto-RGD	188 ± 16 (NR) MBq	90 min	SUV_mean_; TBR	Common carotid artery
Jenkins et al. (19)	Siemens Biograph mCT	^18^F-fluciclatide	229 ± 12 (NR) MBq	40 min	SUV_mean_; SUV_max_; TBR	Superior vena cava
Jenkins et al. (19)	Siemens Biograph mCT	^18^F-fluciclatide	229 (NR) MBq	40 min	SUV_mean_; SUV_max_; TBR	Superior vena cava
Makowski et al. (20)	Siemens Biograph Sensation 16	^18^F-Galacto-RGD	188 ± 19 (NR) MBq	120 min	SUVs; SUV ratios	Normal myocardium, blood, liver
Dietz et al. (21)	GE Discovery 690 TOF; Siemens Biograph Vision 600	^68^Ga-NODAGA-RGD	190 (NR) MBq	63 min	SUV_max_; SUV_mean_; TBR	Inferior and superior vena cava

*NR, not reported; SUV, standardized uptake value; TBR, target-to-background ratio*.

**Table 3:** Summary of PET findings for studies focused on the infarcted myocardium.

**References**	**MI type**	**RGD-based tracer accumulation**				**RGD-based tracer changes according to time after the event**	**Correlation between RGD uptake and MI size**	**Other modalities used to determine the infarcted zones**	**Uptake pattern**	**Histological correlates**
		**Days after MI**	**Positive number**	**SUV values**	**Negative number**					
Sun et al. (16)	NR (one NSTEMI patient specified among the negative patients)	3 days−2 years	20/23	SUV_peak_ 1.94 ± 0.48, SUV ratios 2.33 ± 1.04, with peak uptake 1 week after MI	3	No serial assessment. Peak uptake 1 week after MI. Plateau within 4–75 days after MI	Yes (*r* = 0.748, *P* = 0.001)	^99m^Tc-MIBI/cardiac ^18^F-FDG PET/CT	Patchy form, within the area of infarction or immediately around	NR
Jenkins et al. (19)	STEMI	14 ± 7 days (*n* = 21)	21	TBR_mean_ 1.34 ± 0.22	0	Reduced intensity on second PET/CT scans	No (*r* = 0.03, *P* = 0.90)	Cardiac MRI	Within the area of infarction	Viable myocardium with widespread positive staining for α_v_β_3_ integrin (exploratory analysis for two patients)
		76 ± 19 days (*n* = 17)	17	TBR_mean_ 1.20 ± 0.21	0					
Makowski et al. (20)	7 STEMI/5 NSTEMI	31 ± 14 days	5	Lesion/blood 1.15 ± 0.06; lesion/liver 0.61 ± 0.18	7	NR	Yes (moderate, *r* = 0.73, *P* = 0.016)	Cardiac MRI/[^13^N]NH3 PET	Within the area of infarction or immediately around	NR

*NR, not reported; MI, myocardial infarction; STEMI, ST-elevation myocardial infarction; NSTEMI, Non-ST-elevation myocardial infarction.*

*r = Pearson correlation analysis*.

**Table 4:** Summary of PET findings for studies focused on atherosclerosis.

**References**	**Arterial segment**	**RGD-based tracer accumulation**			**Clinical correlates**	**Morphological imaging correlates**	**Histological correlates**
		**Values; median (IQR)**	**Positive number**	**Negative number**			
Beer et al. (17)	Internal and common carotid arteries	NR	In 5 patients (50%)	In 5 patients (50%)	NR	Higher tracer accumulation in areas of the carotids with medium- or high-grade stenosis compared with areas with none/low-grade stenosis (stenosis classified by using ultrasound and MR angiography, *P* = 0.04).	α_v_β_3_ expression (*r* = 0.787, *P* = 0.026)
Jenkins et al. (19)	Ascending aorta, aortic arch, and descending thoracic aorta (for *in vivo* imaging)	SUV_mean_ 2.73 (2.35–3.05) SUVmax 3.65 (3.04–4.01) TBR 1.31 (1.20–1.39)	NR	NR	Higher tracer accumulation in patients with recent MI (*P* = 0.02), with hypercholesterolaemia (*P* = 0.01) and with established ischemic heart disease (*P* = 0.04)	CT plaque burden (*r* = 0.37, P=0.01), mean wall thickness (*r* = 0.57, *P* < 0.001), and plaque volume (*r* = 0.56, *P* < 0.001) recorded on CT	Tracer accumulation co-localized with areas of α_v_β_3_ expression, angiogenic endothelial cells, and inflammatory macrophages (in four human carotid intimal samples)
Dietz et al. (21)	Common carotid arteries, ascending aorta, aortic arch, descending aorta, abdominal aorta, and iliac arteries	TBR 1.84 (1.62–2.04)	NR	NR	Higher tracer accumulation in patients with previous clinically documented atherosclerotic cardiovascular disease (*P* = 0.001). Positive correlation with prior cardiovascular or cerebrovascular event (*r* = 0.33, *P* = 0.027), BMI (ρ = 0.38, *P* = 0.01), and history of hypercholesterolemia (*r* = 0.31, *P* = 0.04).	CT plaque burden (ρ = 0.31, *P* = 0.04)	NR

*NR, not reported.*

*r = Pearson correlation analysis.*

*ρ = Spearman correlation analysis*.

**Table 5:** Summary of PET findings for studies focused on stroke.

**References**	**Explicit neurologic symptoms**	**RGD-based tracer accumulation**				**RGD-based tracer changes according to time after the event**	**Correlation between RGD uptake and stroke size**	**Other modalities used to determine the infarct zones**	**Uptake pattern**	**Histological correlates**
		**Days after the event**	**Positive number**	**SUV values**	**Negative number**					
Choi et al. (22)	13/17 (per lesion analysis)	1–422 days	8/17 (per lesion analysis)	No SUV value reported, lesion to control ratios 3.9 ± 4.09	9/17 (per lesion analysis)	No serial assessment. Higher tracer accumulation in the recent infarct group (<30 days after the event)	NR	Brain MRI/^99m^Tc-HMPAO perfusion	Higher tracer accumulation in the three lesions with hyperperfusion as compared with the other lesions (*P* = 0.033)	NR
Sun et al. (16)	16/16 patients	4–13 years (*n* = 16)	8/16	SUV_peak_ 0.46 ± 0.29, SUV ratios 3.29 ± 1.09, with peak uptake 2 weeks after the event	8/16	Reduced intensity (*n* = 1) or disappearance (*n* = 1) on second PET/CT scans	In patients scanned 13th−26 days after the event (*r* = 0.835, *P* = 0.003)	Brain MRI/brain ^18^F-FDG PET/CT	Punctate multifocal form	NR
		3 months (*n* = 2)	1/2	SUV_peak_ 0.16 (*n* = 1)	1/2			Brain ^18^F-FDG PET/CT		

*NR, not reported*.

### Qualitative Synthesis (Systematic Review)

#### Basic Study and Patient Characteristics

Through the comprehensive computer literature search, 7 full-text articles including data on the role of RGD-based PET agents in patients with cardiovascular diseases were selected (Table 1) (16–22). All the selected articles were published in the last 9 years. Korea, the United Kingdom, China, Germany, and Switzerland were represented. All the studies were monocentric, 86% were prospective and 14% were retrospective. Two out of the seven studies (29%) focused only on the infarcted myocardium, one study focused on the infarcted myocardium and stroke (14%), one study focused only on stroke (14%), and the remaining 3 studies (43%) focused on atherosclerosis. Included patients had high rates of cardiovascular diseases: 130 out of the 198 patients (66%) had previous MI or stroke or were scheduled for carotid endarterectomy. The number of patients performing PET with RGD-based PET agents in cardiovascular studies ranged from 10 to 46. The mean age of the included patients ranged from 6.1 to 68.5 years; the female percentage was variable from 0 to 50%.

The quality of all the selected studies is judged as moderate according to the NIH quality assessment tools (see Supplementary Figure 1).

#### Technical Aspects

The included studies had heterogeneous technical aspects (Table 2). Five different radiotracers were used, the most frequent being ^18^F-Galacto-RGD and ^18^F-fluciclatide. The hybrid imaging modality was PET/CT for all the studies (no PET/MR study recorded). The radiopharmaceutical injected activity varied among the included studies (Table 2). The mean time interval between radiotracer injection and image acquisition varied among the included studies, from 20 to 120 min. Fasting was not requested before radiolabelled RGD-based injection. The PET image analysis was performed by using qualitative (visual) analysis and additional semi-quantitative analysis through the calculation of standardized uptake values (SUV) in most studies (6 out of the 7 studies: 86%). All reported values were corrected for mean radiotracer activities in the normal myocardium, and/or the normal brain, the blood, the liver, the most frequent being the blood pool activity.

#### Main Findings for Studies Focused on the Infarcted Myocardium

Twenty-nine out of the 56 (52%) MI patients in studies focused on the infarcted myocardium had ST-elevation MI (STEMI), 6 out of the 56 (11%) MI patients had non-ST-elevation MI (NSTEMI), and this information was not available in the 21 remaining patients (38%). The main outcome measurements of RGD-based tracer accumulation in the infarcted myocardium are listed in Table 3 and include correlation between RGD uptake and MI size, characterization of RGD uptake pattern, histological correlates, and the temporal expression of RGD uptake. The time interval between MI and RGD-based PET/CT varied from 3 days to 2 years, most patients undergoing RGD-based PET/CT within 3 months after the MI attack. Importantly, 63 out of the 73 RGD-based PET acquisitions (86%) displayed positive radiotracer uptake in the infarcted zones. Other modalities used to outline the infarcted zones were ^99m^Tc-MIBI, cardiac MRI and [^13^N]NH3 PET.

The MI size was determined by ^99m^Tc-MIBI cardiac perfusion imaging (measurements of the maximum diameters of the infarcted regions), cardiac MRI (measurements in g/m^2^) or [^13^N]NH3 perfusion PET (% of left ventricle). Two out of the three studies found correlation between RGD uptake and MI size but the remaining third study did not find any correlation.

Concerning the correlation between perfusion and RGD uptake, Makowski et al. found a per-segment negative correlation between tracer uptake of [^18^F]Galacto-RGD and blood flow, as measured by [^13^N]NH3 (20). Interestingly, Sun et al. found ^68^Ga-PRGD2 uptake to be located at or immediately around the area of infarction outlined by matched ^99m^Tc-MIBI perfusion images and ^18^F-FDG metabolism images (16). This finding supports a potential increased α_v_β_3_ expression also in non-viable tissue, as defined by regional reductions in ^18^F-FDG uptake in proportion to regional reductions in myocardial perfusion.

Only 1 out of the 3 studies reported histopathological analysis in an exploratory analysis for two patients, finding positive staining for α_v_β_3_ integrin, largely in regions that co-localized to vascular endothelial cells. Lower numbers of CD68-positive inflammatory cells and smooth muscle actin-positive myofibroblasts co-registered also with α_v_β_3_ integrin expression were found in their exploratory analysis (18). One out of the 3 studies studied the temporal expression of the RGD uptake using serial assessments, with 17 out of the 21 patients agreeing to return for a second RGD PET/CT 76 ± 19 days post-MI and found a reduced RGD uptake intensity as compared with earlier imaging 14 ± 7 days post-MI (18).

The study from Jenkins et al. was also the only study that assessed the correlation between RGD uptake and functional recovery. Interestingly, they found an association between increased α_v_β_3_ expression and improvement of wall motion (18).

**Figure 2:**
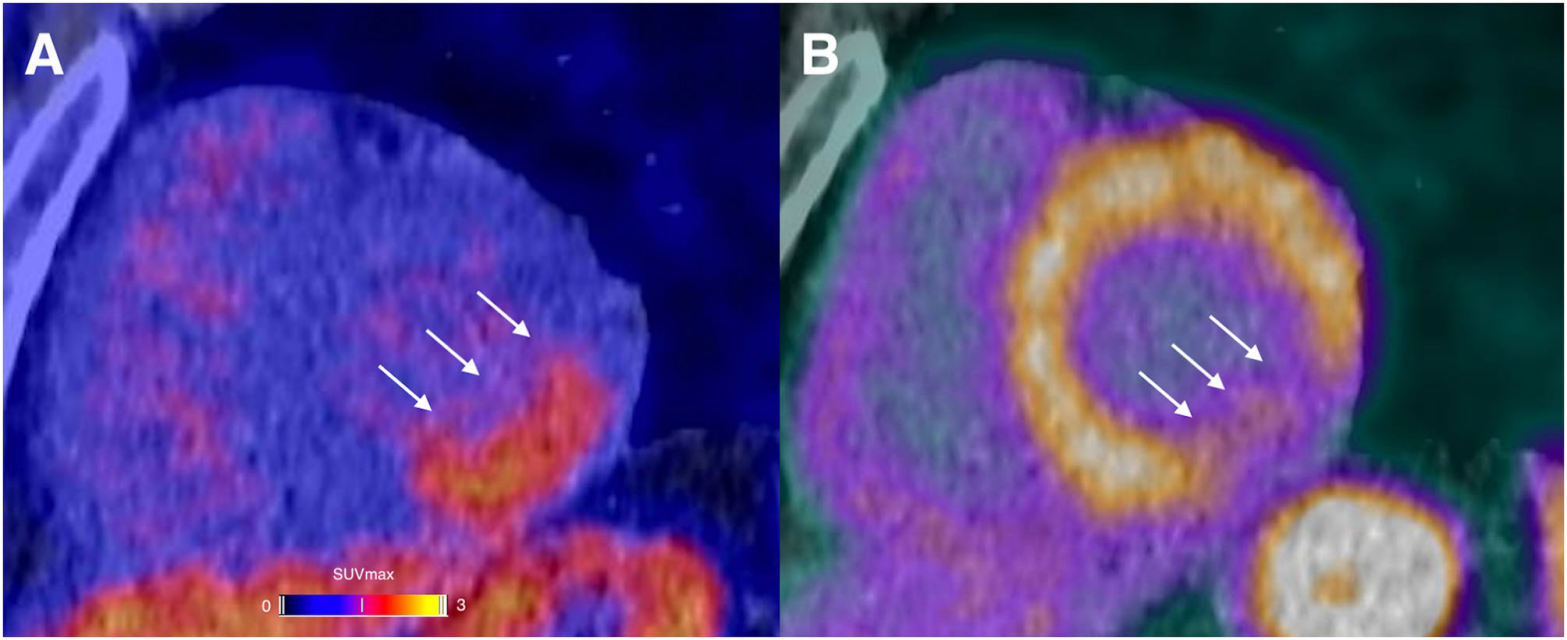
A 44-year-old male presented with chest pain in the emergency room. The initial ECG displayed sinus-rhythm with ST elevation in inferior derivations. The laboratory results showed severely elevated cardiac troponin (up to 7,220 ng/l). Percutaneous coronary intervention with stenting of the completely occluded proximal left circumflex artery was performed without complications. A α_v_β_3_-targeting PET agent (^68^Ga-Nodaga-RGD) was used to assess integrin expression 9 days after the event. Focal tracer retention was localized in the basal and mid inferolateral segments (arrows; **A**). PET/CT examination using Rubidium-82 revealed a severely reduced myocardial blood flow in the same basal and mid inferolateral segments (arrows; **B**).

A representative example of RGD PET/CT imaging in the infarcted myocardium is illustrated in Figure 2.

#### Main Findings for Studies Focused on Atherosclerosis

The arterial segments assessed were internal and common carotid arteries, ascending aorta, aortic arch, descending aorta, abdominal aorta, and iliac arteries. One study focused only on internal and common carotid arteries and another study focused only on thoracic aorta for *in vivo* imaging (17, 19). No study assessed the coronary arteries. The main outcome measurements of RGD-based tracer accumulation in atherosclerosis are listed in Table 4 and include clinical, morphological, and histological correlates. The median intensity of tracer uptake as determined by TBR measurements ranged from 1.31 (IQR 1.62–2.04) to 1.84 (IQR 1.62–2.04), but these data were available only in 2 out of 3 studies (19, 21). Only one study included a binary visual analysis and found a positive signal in carotid plaques on PET using ^68^Ga-NOTA-PRGD2 in only half of the patients (5 out of 10 patients) (17).

Two studies focused on clinical correlates and found correlation with prior cardiovascular or cerebrovascular events as well as with history of hypercholesterolemia (19, 21).

Morphological imaging was included in all studies. Two studies focused on CT plaque burden in large vessels and found correlation with CT calcium scoring and/or the indexed plaque volume (19, 21). One study included further exploration using ultrasound and MR angiography and found a higher tracer accumulation in areas of the carotids with medium- or high-grade stenosis compared with areas with none/low-grade stenosis (17).

Finally, 2 out of the 3 studies reported histopathological analysis and found a co-localization between tracer accumulation and areas of α_v_β_3_ expression (17, 19).

**Figure 3:**
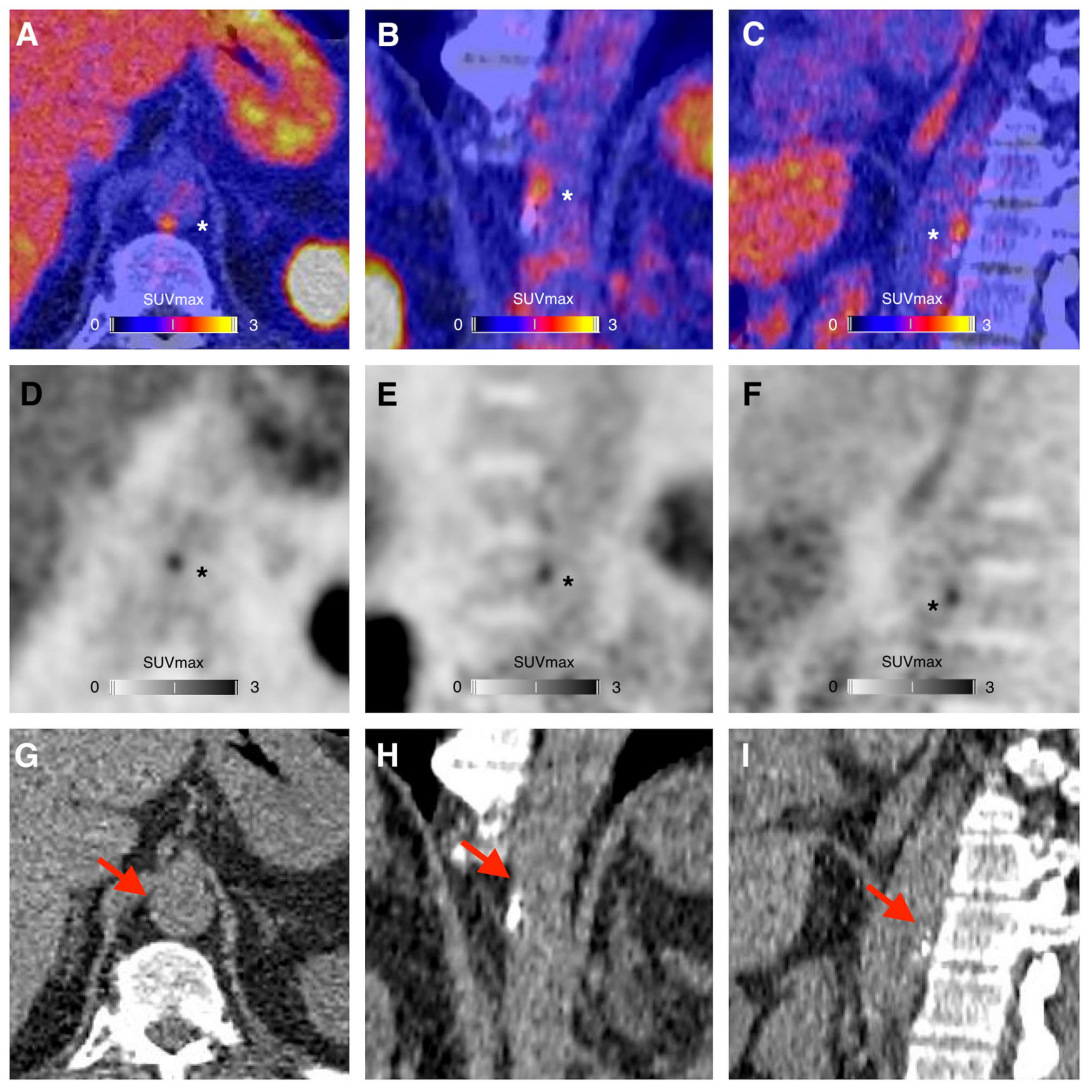
PET/CT **(A–C)**, PET **(D–F)**, and CT **(G–I)** images showing a focus of ^68^Ga-NODAGA-RGD arterial uptake (asterisks), in the periphery of a calcified atherosclerotic lesion (red arrows), in the wall of the abdominal aorta of a 63-year-old man who had a myocardial infarction with ST elevation 3 months before PET imaging. The risk factors of the patient were resumed to current tobacco use and family history of early coronary artery disease. At the time of the PET imaging the patient was treated with high intensity statin therapy, aspirin, ticagrelor, beta-blockers and an angiotensin-converting enzyme inhibitor, and was advised to adopt a healthy diet and to engage in regular physical activity.

A representative example of RGD PET/CT imaging in atherosclerosis is illustrated in Figure 3.

#### Main Findings for Studies Focused on Stroke

The stroke symptoms were described as hemiplegia, hemidysesthesia and/or hemianopsia in the 16 stroke patients included in the study from Sun et al. (16) and were not described in the 10 stroke patients included in the study from Choi et al. (22). The main outcome measures about the RGD-based tracer accumulation in cerebral infarct are listed in Table 5 and include correlation between RGD uptake and stroke size, characterization of RGD uptake pattern, and the temporal expression of RGD. The time interval between the event and RGD-based PET/CT varied from 1 day to 4 years, with most patients within 1 month after the cerebral attack. On a per-lesion analysis, 16 out of the 33 cerebral infarct zones (48%) displayed positive radiotracer uptake. Seventeen out of the thirty-three cerebral infarct zones (52%) were negative. Other modalities used to determine the infarct zones were ^99m^Tc-HMPAO, brain MRI and ^18^F-FDG PET/CT. Only one study studied the correlation between RGD uptake and stroke size, measured over the CT images by referring to the ^18^F-FDG images (16). They found a positive correlation between RGD uptake and infarct size in a limited number of patients (*n* = 10) scanned 13–26 days after the event (Table 5). However, the authors pointed out two patients scanned 4 days after the event with mildly elevated RGD uptake despite relatively large low-density stroke regions on CT (SUV_peak_ = 0.15 and 0.16, maximum diameters 69.8 and 101.3 mm, respectively). No other data about the correlation between RGD uptake and stroke size was reported. This same study described the uptake pattern as a punctate multifocal form, mainly along the surrounding blood vessels (16). These authors also assessed the temporal expression of the RGD uptake using serial assessments with repeated scans at 3 months post event, but with a very limited number of patients (*n* = 2). They found a reduced intensity (*n* = 1) or disappearance (*n* = 1) on second PET/CT scans.

## Discussion

Since the first successful clinical applications of an RGD PET radiopharmaceutical two decades ago, all the final eligible articles included in this review (*n* = 7) were published in the last 9 years, reflecting a recent growing interest in RGD PET/CT imaging focused on cardiovascular diseases (16–22). These studies varied in size and methodology. Two main topics can be distinguished: the infarcted myocardium and atherosclerosis. Stroke is a third topic that can be distinguished, but slightly less studied. All studies had a relatively clearly stated, clinically relevant, and patient-related purpose. Longitudinal studies that tell more about the natural history of α_v_β_3_ expression in cardiovascular diseases using PET imaging were in very short supply. Interventional studies or randomized controlled trials have not been conducted so far. Due to this diversity, our systematic review could not provide an answer to a particular scientific question; we were left to summarize the main information about RGD PET in cardiovascular diseases that the literature had provided until now and point to some major issues that remain unanswered.

### Infarcted Myocardium and α_v_β_3_ Integrin Expression

Ventricular remodeling defines the changes that occur in structure, geometry, and function of the myocardium after MI. This biological process involves inflammation, angiogenesis, repair, and healing with specific biochemical and structural alterations in the infarcted myocardium, peri infarcted and remote regions. Several cell populations are involved, such as neutrophils, monocytes/macrophages, fibroblasts, T cells, stem cells, etc. (23, 24). Understanding reparative mechanisms following MI is becoming increasingly important. In some circumstances, maladaptive persistent processes may be detrimental. This may encourage remodeling and scarring to extend into the myocardium long after the initial causative injury, leading to progressive ventricular dilatation, ventricular dysfunction, and heart failure (25, 26).

The expression of α_v_β_3_ integrin following MI, using PET imaging technique, could be of great interest. In contrast to a low level of expression by quiescent endothelial cells, α_v_β_3_ integrin was found to be upregulated in state of angiogenesis within the myocardium after infarction in preclinical studies in rats (13, 14). α_v_β_3_ integrin expression was also documented by both activated cardiac myofibroblasts and macrophages during margination and chemotaxis. Thus, α_v_β_3_ integrin expression could play a central role in the coordination of the repair processes following MI.

### Prevalence and Natural History of RGD PET Uptake in the Infarcted Myocardium, and Future Potential Directions

Forty-six out of the 56 (90%) MI patients in PET studies focused on the infarcted myocardium presented RGD uptake in the infarcted area, demonstrating that α_v_β_3_ integrin expression in the infarcted myocardium is well evident in RGD PET/CT scans.

In human studies of acute MI, RGD-based PET radiotracers accumulate at the site of infarction as early as 3 days and seem to be peaking at 1–3 weeks post-MI before being decreasing (16, 18). However, only one study assessed serial changes of myocardial RGD uptake after ischemic intervention (18). Moreover, the timepoint of the second ^18^F-fluciclatide PET/CT after MI in this single study was particularly variable (76 ± 19 days). Changes of myocardial RGD PET uptake in humans after coronary occlusion and reperfusion is still to be precisely determined. Serial changes of myocardial RGD PET uptake are studied in more detail in an ongoing prospective study (ClinicalTrial.gov NCT03809689).

Vessel damage with ischemic insult is expected to occur during the first days after MI (27, 28), which could lead to unspecific tracer diffusion in damaged leaky vessels. Here, the peak of tracer accumulation at 1–3 weeks post-MI is in favor of minimal effects of this unspecific tracer diffusion in damaged leaky vessels. Myocardial uptake post MI using RGD PET/CT may thus be predominantly due to integrin expression rather than unspecific leakage. However, no imaging was performed at day 1 in the different studies.

An interesting finding in both studies from Sun et al. (16) and Jenkins et al. (18) was the absence of increase in RGD uptake in chronically damaged myocardium, indicating minimal residual α_v_β_3_ integrin expression in old ischemic injuries. Therefore, α_v_β_3_ integrin expression seems rather to act as a marker of cardiac repair at sites of recent MI (16, 18).

Despite those promising results further prospective studies are nevertheless warranted to confirm a clinical value of RGD PET uptake as a prognostic marker for left ventricular remodeling. Moreover, some findings support increased α_v_β_3_ expression also in non-viable tissue, which could be explain by a linking to the myofibroblasts (16, 29). Studies assessing the prognostic value of RGD PET uptake in the infarcted myocardium are still needed.

### Atherosclerosis and α_v_β_3_ Integrin Expression

The ability to accurately and non-invasively monitor inflammatory processes in atherosclerotic plaques would represent a major advance, and metabolic imaging using PET has the potential to fulfill this important unmet clinical need. Integrin α_v_β_3_ has been widely studied in atherosclerosis. In the development of atherosclerosis, expression of integrin α_v_β_3_ has been found in endothelial cells as well as in CD68-positive macrophages, and both inflammation and angiogenesis processes are associated with plaque growth, plaque instability and clinical events (10–12). The necrotic core in the culprit plaque results from increasing inflammation (30). Pro-atherogenic stimuli lead to an infiltration of activated monocytes within the intima, which differentiate into pro-inflammatory macrophages (31). The apoptosis of the resident pro-inflammatory macrophages leads to the development of a lipid-rich or necrotic core (30). Matrix metalloproteinases secreted by macrophages weaken the fibrous cap, predisposing it to rupture. Angiogenesis is believed to occur in response to hypoxic conditions within the necrotic core. Neovessels, arising from the adventitial vasa vasorum, grow into the base of progressive atherosclerotic lesions and provide an alternative entry pathway for monocytes and immune cells. The plaque neovessels are fragile and leaky, giving rise to local extravasation of plasma proteins and erythrocytes (32). Plaque hemorrhage itself results in a pro-inflammatory response, plaque destabilization and clinical events (11, 33).

### Attractive Promising Results of RGD-Based PET Agents in Atherosclerosis

Overall, although the numbers are limited, the existing studies support the potential of RGD-based PET imaging of α_v_β_3_ integrin expression in atherosclerosis. ^18^F-fluciclatide PET/CT has proven itself as a potential valuable target for the imaging of unstable atherosclerosis (19). ^68^Ga-NODAGA-RGD uptake in large vessels showed clinical correlation with atherosclerotic cardiovascular diseases (21). ^18^F-Galacto-RGD PET/CT showed specific tracer accumulation in human atherosclerotic carotid plaques, with increased PET signals observed in stenotic compared with non-stenotic areas based on MR or CT angiography data, and with histological correlation with α_v_β_3_ expression (17).

### Intensity of RGD PET Uptake in Plaque Imaging in Humans

Background corrections were systematically used among different studies. Again, the median intensity of arterial tracer uptake as determined by TBR measurements were 1.31 (IQR 1.20–1.39) for the study from Jenkins et al. (19) and 1.84 (IQR 1.62–2.04) for the study from Dietz et al. (21). Interestingly, Dietz et al. used a SiPM PET system (Biograph Vision 600, Siemens Medical Solutions, Knoxville, USA) in 16 out of 44 (36%) of the patients included for the analysis, which could partly explain the highest TBR measurements. SiPM PET is a new PET technology with improved spatial and timing resolution and a relatively high sensitivity and count-rate capability as compared to PET scanners using conventional photomultiplier tubes (34).

The TBR measurements were slightly lower than those of other established tracers used for plaque imaging. In a study using ^18^F-sodium fluoride for visualization of microcalcification in plaque in the major arteries, the mean TBR was 2.3 ± 0.7 (35). In another study using ^68^Ga-Pentixafor for visualization of inflammatory cells, the mean TBR was 2.0 ± 0.5 (36).

The difference in intensity of TBR between non-calcified/mixed/calcified plaques on CT has not been studied so far. However, Dietz et al. found that only around 26% of ^68^Ga-NODAGA-RGD highest uptake foci in their cohort were colocalized to calcification on CT (21). This result suggests that RGD accumulation could particularly occur mainly in the non-calcified vessel wall, which may indicate some association with pathophysiologic processes found in early atherosclerotic disease.

### Future Potential Directions of α_v_β_3_ Integrin Expression in Plaque Imaging

To date, RGD PET imaging in humans has emerged as a promising tool for the assessment of active atherosclerosis processes, but with a still very limited number of studies (*n* = 3) (17, 19, 21). All studies were focused on subjects with established disease, and there is no work which has investigated whether RGD uptake could predict future events or whether it can provide additional prognostic information over current methods of risk stratification. Further works are also needed to establish whether RGD PET could monitor disease progression, guide therapeutic interventions, or assess novel anti-angiogenic therapies in cardiovascular diseases.

It is also not well established whether having a recent MI would lead to a significant increase in aortic plaque microvessels, or indeed whether these changes are likely to occur within 1–3 weeks post MI. Because integrins α_v_β_3_ are expressed in neovessels in plaques but also in CD68-positive macrophages, it is not possible to ascertain with PET only whether RGD is binding preferentially to one or the other of these processes. Aortic RGD uptake post MI in large vessels could be reporting predominantly on systemic inflammation rather than microvessels proliferation (37).

No study assessed the coronary arteries. The potential value of integrin tracers would be of great interest for future trials, as most PET tracers are not suitable due to too low signal for coronary artery disease assessment. Moreover, the capacity of the paradigm based on ischemia to prevent MI is increasingly being questioned, and the quantification of metabolic activity of atherosclerotic disease is of growing interest (38). PET imaging with integrin tracers could have the potential to add value to this purpose.

No PET study in humans assessed α_v_β_3_ integrin expression in aortic aneurysm, which could be of clinical interest. Degraded aortic tissue is known to exhibit abnormal angiogenesis and is present in abundance at sites of aortic rupture (39). α_v_β_3_ integrin expression stimulate angiogenesis, typically in hypoxic environments (40). The predictive value of α_v_β_3_ integrin expression imaging using PET in aortopathy to predict disease progression is unknown.

### RGD PET Uptake in Stroke

PET studies focused on ischemic cerebral vascular disease are scarce (*n* = 2) (16, 22). As in the normal myocardium, the PET RGD-peptide was absent in the normal brain, allowing a clear background to evaluate the post-stroke angiogenesis. However, the RGD PET uptake levels in stroke seem to be lower than those in MI (16). These could indicate difficulties of forming new capillaries from the pre-existing vessels after stroke (16). The existing data support that α_v_β_3_ integrin expression in the cerebral infarct is prominent in the acute phase (<30 days after the event) and decrease with time, but evidence is still lacking. Serial changes of cerebral RGD uptake were assessed for only two patients (16). However, both existing studies have consistent findings in chronically damaged brain, with absent or very weak uptake reported.

Some evidence supports a strong correlation between RGD uptake and stroke size but only in a limited number of patients scanned 13–26 days after the event (16). Like in MI, a non-perfect correlation between RGD uptake and standard measures of infarct severity like the infarct size could suggest that RGD uptake is not a surrogate of infarction but could relate more to the tissue-healing response to injury.

An interesting finding in the study from Sun et al. is a punctate multifocal RGD uptake along the surrounding blood vessels in stroke patients, contrasting with the patchy-form uptake found in MI patients (16). The authors explained that the myocardial remodeling could contribute to this different uptake pattern, but more data are needed to confirm these findings.

### Radiolabeling of RGD-Based PET Agents

Most of the RGD-based radiotracers included in this systematic review were labeled with ^18^F (4 out of the 7 studies: 57%). The remaining 2 out of the 7 studies (29%) used RGD-based radiotracers labeled with ^68^Ga obtained from a ^68^Ge/^68^Ga generator. Imaging relatively thick structure like the left ventricle is not an issue using PET imaging but the use of ^18^F labeled tracers for atherosclerotic PET imaging could have several physical and technical advantages as compared to ^68^Ga labeled tracers. In fact, ^68^Ga has higher positron energy and positron range which could lead to noisier images and worse spatial resolution, which could be amplified by a lower injected activity of ^68^Ga compared with ^18^F (at least using traditional photomultiplier tube PET/CT). However, the synthesis of ^18^F-Galacto-RGD tracer used by Beer et al. could be complex and time consuming, making an automated production process, which is mandatory for routine clinical use, extremely difficult (17, 41, 42). Moreover, ^68^Ga labeled tracers have already been extensively used to image atherosclerotic plaques in large vessels, and in vessels as small as the coronary arteries (36, 43, 44). The superiority of a labeling with ^18^F or ^68^Ga is not clear for RGD-based radiotracers for cardiovascular PET imaging.

### Limitations

We should report several limitations in our systematic review, which could limit the scope of our results. Our eligibility criteria lead to a very small number of original articles eligible for inclusion in this systematic review, with limited histological assessment. This systematic review focused on PET, and studies focusing on SPECT were not included. Unlike SPECT imaging, PET imaging does not require an extrinsic collimator resulting in higher count sensitivity and spatial resolution. Moreover, accurate and well validated attenuation correction is available with PET. This provides routinely available quantitative assessments (45), which could be crucial. In fact, important potential clinical applications of molecular integrin imaging in cardiovascular diseases could provide quantitative endpoints for use in clinical trials.

There was significant heterogeneity across the studies in terms of technical aspects. Different radiotracers were used. The radiopharmaceutical injected activity and the mean time interval between radiotracer injection and image acquisition varied among the included studies.

For atherosclerosis imaging, it cannot be ruled out that tracer signals observed with RGD PET are at least partially attributable to noise, especially using ^68^Ga labeling (46).

Finally, despite a high number of prospective studies with relatively clearly stated and patient-related purposes, according to the NIH quality assessment tool the quality of the seven selected studies is judged as moderate, mainly as they were single center and with a limited number of patients. We have tried to minimize publication bias excluding case reports or small case series from this systematic review.

## Conclusions

From this systematic review on the role of RGD-based PET agents in patients with cardiovascular diseases, we are led to conclude that data on PET imaging of integrin α_v_β_3_ in patients with cardiovascular diseases are still very limited. PET imaging can provide insight on unique markers of disease activity, such as neoangiogenesis and inflammation, which are crucial to the pathogenesis of cardiovascular diseases. Promising applications using RGD-based PET agents are emerging, such as prediction of remodeling processes in the infarcted myocardium or detection of active atherosclerosis, with potentially significant clinical impact.

## Data Availability Statement

The raw data supporting the conclusions of this article will be made available by the authors, without undue reservation.

## Author Contributions

MD and GT: conceptualization and investigation. GT: methodology. MD: writing—original draft preparation. MD, CK, VD, SF, VR, NT, AS, MJ, SB, MN, NS, NM, JP, and GT: writing—review and editing. JP and GT: supervision. All authors have read and agreed to the published version of the manuscript.

## Funding

MD was supported by Research Fellowship Awards from the Société Française de Radiologie, Paris, France, and from the Agence Régionale de Santé Auvergne-Rhone-Alpes, Lyon, France. Open access funding was provided by the University of Lausanne.

## Conflict of Interest

The authors declare that the research was conducted in the absence of any commercial or financial relationships that could be construed as a potential conflict of interest.

## Publisher's Note

All claims expressed in this article are solely those of the authors and do not necessarily represent those of their affiliated organizations, or those of the publisher, the editors and the reviewers. Any product that may be evaluated in this article, or claim that may be made by its manufacturer, is not guaranteed or endorsed by the publisher.
