# Erratum: Overview of the RGD-based PET agents use in patients with cardiovascular diseases: A systematic review

**DOI:** 10.3389/fmed.2023.1183711

**Published:** 2023-03-22

**Authors:** Frontiers Production Office

**Affiliations:** Frontiers Media SA, Lausanne, Switzerland

**Keywords:** αvβ_3_ integrin, RGD, angiogenesis, positron emission tomography, cardiovascular diseases, myocardial infarction, atherosclerosis

An omission to the funding section of the original article was made in error. The following sentence has been added: “Open access funding was provided by the University of Lausanne.”

The original article has been updated.

