# Supplementary material for: Overview of the RGD-Based PET Agents Use in Patients With Cardiovascular Diseases: A Systematic Review
Source: Front Med (Lausanne). 2022 May 6;9:887508. doi: 10.3389/fmed.2022.887508 (PMC9120643; doi:10.3389/fmed.2022.887508)
Supplement: Supplementary file 1 [file Data_Sheet_1.docx]

Supplementary Figures


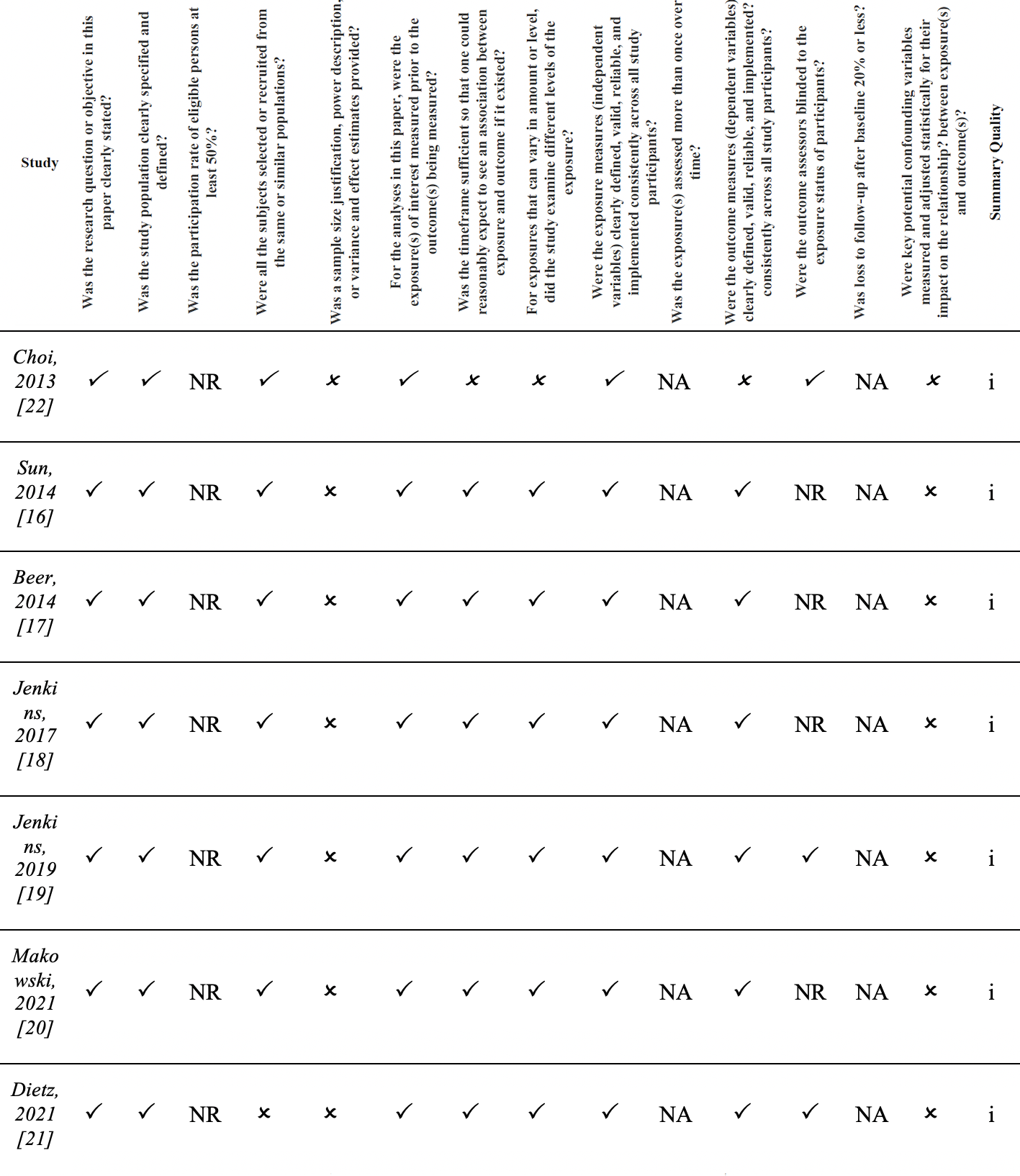


**Supplementary Figure 1.** NIH quality assessment tool. Quality was rated as 0 for poor (0–4 out of 14 questions), i for fair (5–10 out of 14 questions), or ii for good (11–14 out of 14 questions); NA: not applicable, NR: not reported.
